# The relevance of the interpersonal theory of suicide for predicting past-year and lifetime suicidality in autistic adults

**DOI:** 10.1186/s13229-022-00495-5

**Published:** 2022-03-21

**Authors:** R. L. Moseley, N. J. Gregory, P. Smith, C. Allison, S. Cassidy, S. Baron-Cohen

**Affiliations:** 1 Department of Psychology, Bournemouth University, Talbot Campus, Fern Barrow, Poole, BH12 5BB Dorset UK; 2 Autism Research Centre, Department of Psychiatry, University of Cambridge, Cambridge, UK; 3 School of Psychology, University of Nottingham, Nottingham, UK

**Keywords:** Suicide, Thwarted belongingness, Perceived burdensomeness, Acquired capability, Relationships, Age at diagnosis

## Abstract

**Background:**

While there are known risk factors for suicidality in autistic adults, these are often unconnected from theoretical frameworks that might explain *why* risk is elevated and guide clinical interventions. The present study investigated the relevance of constructs from the Interpersonal Theory of Suicide (ITS), including perceived burdensomeness, thwarted belongingness and acquired capability for suicide, and explored mechanisms through which certain risk factors (relationship status, age at diagnosis) might elevate suicide risk.

**Methods:**

Autistic adults (*n* = 314) completed an online study including measures of depression, anxiety and constructs from the ITS. Linear and multinomial regression analysis disentangled contributions of ITS variables from effects of depression and anxiety for past-year suicide ideation, past-year and lifetime suicide attempts. Mediation analyses examined associations between risk factors and these suicide outcomes via mechanisms proposed by the ITS.

**Results:**

Past-year suicide ideation was associated with burdensomeness, mental rehearsal of suicide plans (a facet of acquired capability), and depression. Greater feelings of burdensomeness, and reduced fear of death, marked out participants who had attempted suicide in comparison to those who had experienced suicide ideation in the past year. Relationship status was indirectly associated with past-year suicide ideation via the mediators of depression and burdensomeness, and was associated with past-year attempts via its effect on ideation. Age at diagnosis was unrelated to any variables.

**Limitations:**

Cross-sectional research is insensitive to causality and temporal dynamics, which is likely why interaction hypotheses from the ITS were unsupported. Normative measures may be invalid in autistic samples. There was no control group. The autistic sample was unrepresentative of the whole population, particularly autistic people with intellectual disabilities, ethnic/racial minorities, and gender minorities.

**Conclusions:**

Perceived burdensomeness and acquired capability appear potentially important to suicide in autistic people, and may mediate the effects of some risk factors. Future research should explore the temporal dynamics of suicide trajectories in longitudinal, prospective designs.

**Supplementary Information:**

The online version contains supplementary material available at 10.1186/s13229-022-00495-5.

Suicide ideation, attempts and deaths are unacceptably high in autistic adults without intellectual disabilities [1–5]. Factors associated with suicide ideation and attempts include loneliness and unmet support needs [6, 7], self-injury [6, 8], psychiatric illnesses and post-traumatic symptoms [4, 9], rumination and low self-worth [10], and the extent to which individuals camouflage their autism [6]. In the general public, older age, educational attainment and employment are associated with reduced suicide risk, but the protective effects of these factors appear attenuated or even absent in autistic people [4]. Though traditionally women have reported to experience greater rates of suicide ideation and lower rates of suicide attempts and deaths than men [11, 12] these trends do not hold in autistic men and women [4, 9, 13] (though see [1]).

Recent years have seen attempts to tether knowledge about suicide in autistic people to existing models about suicide in the general population, which might afford deeper understanding and predictive utility. Particular attention has been paid to the Interpersonal Theory of Suicide (ITS) [14], which holds that “people die by suicide *because they can* and *because they want to*” (p. 583, italics added). Importantly, this distinguishes suicidal ideation and attempts as distinct phenomena with shared and unique correlates, and highlights that only a small percentage who experience the former progress to the latter. With reference to suicide ideation, the ITS asserts the importance of thwarted belongingness, a state of social disconnection, and perceived burdensomeness (henceforth ‘burdensomeness’), wherein the individual feels so irredeemably flawed or worthless such as to be a liability to those around them. While either can produce a passive wish for death, the combination of both together invokes strong and active feelings of suicide ideation. For suicide ideation to progress to attempts, the theory asserts the need for an individual to acquire ‘capability’ for suicide, typically via exposure to physically painful and/or emotionally provocative events, including self-injury, which habituate them to pain and reduce the evolutionary fear of pain and dying.

Intuitively, ITS constructs appear highly relevant to autistic people, given the experiences likely to generate states of thwarted belongingness, burdensomeness, and acquired capability for suicide (henceforth ‘acquired capability’): social ostracism, bullying and victimization [15–17], adverse childhood experiences [18], chronic unemployment [19], self-injury [20, 21], marginalization [22], and traumatic or alienating experiences with the professionals and systems designed to support them [23, 24]. It also seems likely that autistic people might accrue capability for suicide at higher rates than non-autistic people, given the greater frequency of self-injury [25, 26], abuse and assault [17, 27, 28] in autistic children and adults. Differences in the rate at which autistic people acquire capability for suicide might also be posited, since capability for suicide can also be related to genetic or dispositional attributes (such as differences in pain sensitivity, squeamishness and harm avoidance) [29]. Accordingly, differences in the way that autistic people experience pain, their own and that observed in other people [30–32], may also be relevant to the ease and speed at which they might progress from suicide ideation to attempts.

Given the likely relevance of these constructs, two investigations thus far have examined predictions from the ITS in autistic samples. Pelton and colleagues [9] examined thwarted belongingness, burdensomeness, and their interaction as predictors of suicide ideation, and burdensomeness, thwarted belongingness, acquired capability and their interaction term as predictors of suicide attempts. They found that although thwarted belongingness and burdensomeness were higher in autistic participants, these constructs explained less of the variance in suicide ideation and attempts than in the non-autistic comparison group. Whilst in non-autistic people suicide ideation was predicted by thwarted belongingness, burdensomeness, and their interaction, this interaction was a non-significant predictor of suicide ideation in autistic people. In this group, both thwarted belongingness and burdensomeness individually predicted suicide *attempts*, leading the authors to suggest the progression from ideation to attempts might be faster in autistic individuals. Acquired capability predicted suicide attempts in both populations, but was no greater in autistic than non-autistic participants. Dow et al. [33] examined relationships, in autistic adults, between depression, suicide ideation, suicide attempts and a subset of items, taken from standardised scales, which reflected thwarted belongingness, burdensomeness, and acquired capability. They reported a significant relationship between acquired capability and suicide attempts, and a relationship between burdensomeness and suicide attempts. Both thwarted belongingness and burdensomeness, in this study, predicted suicide ideation; furthermore, both were associated with lifetime history of depression.

While both studies reflect important investigations into the relevance of ITS constructs to autistic people, there is need for further investigation on several fronts. Firstly, neither of these previous studies were able to examine a fundamental argument of the ITS: that thwarted belongingness and burdensomeness, albeit closely related to depression and anxiety, are separate, distinct predictors and necessary antecedents of suicide ideation [14, 34, 35]. As these previous studies did not model depression and anxiety independently, any distinct relevance of ITS constructs in autistic people could not be established. Secondly, only Pelton and colleagues addressed another central tenet of the ITS: that the interaction of *co-occurring* thwarted belongingness and burdensomeness is critical for suicide ideation, over and above main effects of these variables; and, similarly, that the interaction of thwarted belongingness and burdensomeness, co-occurring with a state of acquired capability, is crucial for suicide attempts. The unique importance of these interactions between constructs was not supported in their sample of autistic adults, but this finding requires replication, especially as the authors employed a narrow conceptualization of acquired capability. While they focused solely on reduced fear of death, more recent approaches suggest a three-faceted construct including mental rehearsal of suicidal actions alongside reduced fear of death and greater pain tolerance. Engaging in mental rehearsal, according to George et al. [36], “inject[s] [thoughts of suicide behaviours] with an active, volitional quality” (p. 1460), which crystallizes intent and amplifies ideation in addition to building capability for execution of these actions. There is, as such, a need to examine this variable in greater depth and to reconsider its associations with both suicide ideation and suicide attempts.

Finally, to understand the relevance of ITS constructs for suicide in the autistic community, it is important to explore whether they can *explain*, i.e., whether they are the mediating mechanism, for associations that have been previously reported between suicidality and certain factors relevant to autistic people. Two such features were chosen for investigation here: relationship status and age at diagnosis. In relation to the former, lower rates of suicide attempts and suicide deaths have been observed in autistic people who were in relationships (married or cohabiting) than in those who were single [4]. Given the centrality of social connectedness and reciprocity in the ITS, the ‘living situation’ of participants was indeed formerly highlighted by Pelton et al. [9] as a variable of potential import. Satisfactory intimate relationships can be a source of social connectedness and support [37], and while being in a healthy relationship can support self-esteem, having *ever* begun a relationship is also associated with greater feelings of self-worth [38]. It follows naturally from this that satisfactory relationships might be associated with lower likelihood of states characterised by thwarted belongingness and burdensomeness, and that this mechanism might explain the association observed in the general population between satisfactory relationships and reduced risk of suicide ideation [39].

With reference to age at diagnosis, one recent report suggests a relationship where suicide attempts increased with older age at diagnosis [4]. While not all studies support this association [6], it is consistent with findings of greater incidence of psychiatric illnesses (including depression, anxiety, OCD and eating disorders), self-injury, behavioural and social difficulties in late-diagnosed children and adolescents [40–42]. In adults, one study corroborated that a lifetime history of depression was predictive of older age at diagnosis [43]. Reflecting on their findings, Hosozawa et al. [40] suggested that individuals diagnosed later in childhood are less likely to receive evidence-based interventions and support, and are potentially at greater risk of being bullied. Qualitative work indeed suggests that bullying and victimization is commonly experienced by late-diagnosed autistic adults, who often perceive themselves as inadequate and as outcasts [44–46]. Another potential link between late diagnosis, mental ill-health and suicidality lies in the probability that these individuals may be more adept at camouflaging their difficulties [47], a correlate of older age at diagnosis which is independent of the sex of participants [48]. Camouflaging has previously been associated with suicidality [6], and in non-autistic people, at least, thwarted belongingness mediates this link [49].

To address gaps in previous investigations and extend research in this area, the present study aimed to re-examine the relevance of the ITS and the validity of its hypotheses in relation to suicide ideation and attempts in autistic adults. Our first set of analyses examined ITS constructs as *predictors* of suicidality, both in terms of their independent (main) effects and interaction effects reflecting their co-occurrence. For suicide ideation, the ITS suggests we should expect main effects of thwarted belongingness, burdensomeness and the mental rehearsal aspect of acquired capability, over and above effects of anxiety and depression; it also suggests that the two-way interaction of thwarted belongingness and burdensomeness should explain additional unique variance. For suicide attempts, previous literature in autistic adults [9] led us to expect main effects of thwarted belongingness and burdensomeness, alongside all aspects of acquired capability (as originally proposed by the ITS); the ITS also suggests that additional unique variance should be explained by the interaction term of all three constructs.

Our second set of analyses examined whether thwarted belongingness and perceived burdensomeness *mediate*, or explain, previously reported associations between suicidality and relationship status [4], and between suicidality and older age at diagnosis [4]. Both autism-relevant variables could conceivably be associated with thwarted belongingness and burdensomeness; if these ITS constructs are relevant for suicide ideation and attempts, it follows that they may, therefore, explain associations between relationship status and age at diagnosis and suicidality. In that romantic relationships are often a source of support and can bolster feelings of self-worth [37, 38], being in a relationship at the time of the study was hypothesised to be associated with reduced feelings of thwarted belongingness and burdensomeness, and through this reduced likelihood of suicide ideation and attempts. In that older age at diagnosis might be associated with greater camouflage, reduced support, intolerance from others, and feelings of failure and alienation [40, 41], we hypothesised that it might be associated with stronger feelings of thwarted belongingness and burdensomeness, and influence suicide ideation and attempts via these means.

## Methods

### Participants

Recruitment for the study occurred via three approaches. Firstly, from June to December of 2020, advertisements for the study were posted on social media (Facebook and Twitter communities run by and for autistic people) and distributed to the Cambridge Autism Research Database (CARD: approximately 34.7% of the final sample). During the same period, we also contacted participants from prior studies [8, 21] who had consented to receive news of any future research by our group (22.6% of the final sample). In January 2021, we distributed our advertisement to the volunteer panel of Autistica’s research network (42.7% of the final sample). Altogether, the sample comprised 314 autistic adults, whose demographic details can be seen in Table 1. A formal diagnosis is necessary for inclusion on the CARD database, but this is not a prerequisite for Autistica research volunteers. Although we did not independently verify diagnoses, all participants provided the date and place of their diagnostic assessment and some also reported the precise diagnosis given (if relevant). The majority of participants (95.8%) lived in the UK; a small minority were based in the USA (1.3%) and the remainder hailed from countries within the European Union and South America.

**Table 1:** Demographic details for autistic participants

Sample characteristics (*n* = 314)			
Average age	41.9 years (SD 13.4, range 18–72)		
Average age at diagnosis	34.6 years (SD 14.8, range 2–67)		
Sex	Male: 26.8%	Female: 72.9%	Other: 3%
Gender identity	Cisgender male: 25.2%	Cisgender female: 57.3%	
	Non-binary: 14.6%	Transgender : 2.9%	
Ethnicity	Caucasian/White: 79.9%	Black: 1.6%	Mixed race: 5.4%
	Other ethnicities: 4.3%	No response: 8.8%	
Highest level of educational attainment	GCSEs, high-school diploma or equivalent: 94.9% Bachelors degree: 70.1% Postgraduate qualifications: 35.7%		
Employment, voluntary work, or productive activities*	Employed full time: 24.8%	Employed part-time: 15.3%	
	Freelancing/self-employed: 10.8%	Looking for work: 6.7%	
	Not looking for work: 5.4%	Studying: 14.7%	
	Voluntary roles: 2.5%	Retired: 5.1%	
	Performing care duties (including childcare): 3.8%		
Relationship status	Married/cohabiting: 35.7%	Relationship/dating: 10.2%	
	Divorced, bereaved, separated: 7.3%	Single: 42.9%	
	Single through choice: 1.3%	No response: 2.6%	
Neurodevelopmental conditions*	ADHD/ADD: 17.2%	Dyslexia: 8.9%	
	Dyspraxia: 8.6%	Other specific learning disabilities: 6.4%	
Psychiatric conditions*	Depression: 7.9% Anxiety: 9.6% Depression and anxiety: 39.8%	PTSD/cPTSD: 10.2% Eating disorders: 8.6%	OCD: 8.6% Other: 9.4%
	Single psychiatric condition: 18.2%	Two psychiatric conditions: 27.4%	3 + psychiatric conditions: 23.2%
	No diagnoses: 31.2%	Suspected anxiety, depression, PTSD/cPTSD: 31.4%	

Demographic information for autistic sample. Rows marked with asterisks reflect categories where participants could affirm multiple options

Note that due to a limitation in the way this option was phrased, this description may not fully reflect transgender participants' gender identities-

Sample size calculations were based on Pelton et al. [9], who reported effect sizes (*f* ^2^) of 0.11 (the upper end of small) and 0.18 (medium) in regression analyses for suicide ideation and attempts in autistic people. For the number of predictors in our own models of suicide ideation [5] and attempts [7], with power set at 90% and predicted effect size as small (in order to be conservative), minimum required sample sizes would have been 156 and 174 respectively.

### Materials and procedure

Ethical approval for the study was granted by the Science and Technology Faculty Ethics Panel at Bournemouth University, with data collected between July 2020 and March 2021. Participants completed a number of measures, hosted on Qualtrics, which yielded scores for the following constructs. Internal consistency for these scales is displayed in Table 2, alongside descriptive data for this sample.

**Table 2:** Internal consistency and descriptive statistics across measures

Measures and constructs	Average score (SD), *range*	Suggested clinical cut-offs in non-autistic populations	*α*	
			Previous literature (non-autistic)	Present Sample
*INQ-15*				
Thwarted belongingness	38 (9.2), *10–63*	31	.81–.87	.87
Perceived burdensomeness	17.1 (9.6), *6–42*	22	.85–.90	.93
*ACWRSS*				
Acquired capability total	29.9 (13.2), *0–56*	–	Range from .74–.81	.78
Pain tolerance	6.6 (4.9), *0–16*	–		.78
Reduced fear of death	7.5 (5.2), *9–16*	–		.75
Mental rehearsal of suicide	15.9 (7.6), *0–24*	–		.84
PHQ-9: Depression total	13 (7.2), *0–27*	8	.86–.89	.90
GAD-7: Anxiety total	11 (5.9), *0–21*	10	.92	.91

Scores and internal consistency for subscales and total scale scores used in this analysis. Clinical cut-offs and comparative alpha levels in non-autistic populations are displayed for the INQ-15 [50, 51], ACWRSS [36], PHQ-9 [52, 53], and GAD-7 [54, 55]

#### ITS constructs

##### Thwarted belongingness and perceived burdensomeness

The Interpersonal Needs Questionnaire-15 (INQ-15) [56] was chosen for its psychometric strengths and predictive validity [50]. It includes nine statements related to thwarted belongingness (e.g. “These days, other people care about me”) and six to burdensomeness (e.g. “These days, I think I am a burden on society”). Participants responded to items on a scale from 1 (“Not at all true for me”) to 7 (“Very true to me”), with higher scores indicating greater thwarted belongingness and burdensomeness.

##### Acquired capability

In accordance with their three-factor model of acquired capability, George et al. [36] developed a 7-item scale, the Acquired Capability with Rehearsal for Suicide Scale (ACWRSS). On an 8-point scale between “Not at all” to “Very strongly”, participants responded to two items on pain tolerance (e.g. “I can tolerate pain much more than I used to”), two reverse-scored items on reduced fear of death by suicide (e.g. “Even if I wanted to, killing myself is too scary to follow through with it”), and three items on mental rehearsal (e.g. “I have thought of ways to kill myself that would be the least difficult for me to pull off”), with higher scores reflecting greater acquired capability.

#### Other predictors and covariates

##### Depression and anxiety

The Patient Health Questionnaire-9 (PHQ-9) [52] and the Generalised Anxiety Disorder-7 (GAD-7) [54] are brief screening measures for depression and anxiety which are in popular clinical use. The former has recently been validated for use in autistic people [57], while the GAD-7 has previously shown good internal consistency in autistic samples [58, 59].

#### Outcome variables

##### Suicide ideation

The frequency of suicide ideation within the last 12 months was assessed with a single item (“How many times in the past year have you thought about suicide?”) from the Self-Injurious Thoughts and Behaviours Interview, short form (SITBI) [60]. Participants could respond “Never” (scored 0), “Once or twice a year” (1), “Three or four times a year” (2), “Once or twice a month” (3), “Once or twice a week” (4), “Three or four times a week” (5), or “Almost every day” (6). If participants responded with positive affirmation of suicidal thoughts, they were asked, “When you have thoughts of killing yourself, how long do these usually last?” and could respond with “1–60 s” (scored 1), “2–15 min” (2), “16–60 min” (3), “More than an hour, but less than 1 day” (4), “1–2 days” (5), or “More than 2 days” (6). Because autistic adults may respond to questions about suicide very literally [61], our analyses all employed a composite score which was created from the sum of an individual’s score to the frequency question (1–6) and their score to the duration question (1–6). Aside from participants who received a 0 indicating no suicide ideation within the past year, scores on this composite could range from 2 to 12. Low scores on this composite measure indicated suicide ideation that was rare and/or brief; higher scores indicated the presence of suicide ideation which was very frequent even if brief, very intense even if less frequent, or highly frequent *and* intense.

##### Suicide attempts

To assess lifetime suicide attempts, the SITBI includes an item, “How many times in your lifetime have you made an actual attempt to kill yourself, in which you had at least some intent to die?”; to which participants could respond with “Never” (scored 0), “Once” (1), “Twice” (2), “Three or four times” (3), or “Five or more times” (4). Responses to this question were used as a continuous outcome measure in continuous regression, and to classify participants as having lifetime experience of a suicide attempt in multinomial regression. A follow-up question asked for the recency of their last attempt, and allowed us to categorise participants as having attempted suicide in the last year.

### Ethical considerations

Research in this area must balance scientific rigour with extreme caution and sensitivity, now more than ever in light of global events [62]. Prior to applying for ethical approval, the design of the study was discussed with one Clinical Psychologist at Bournemouth University and two researchers in suicidology (one working with the general population, one with autistic people). A number of steps were taken to ensure the safety and comfort of participants, including mood-mitigation and extensive signposting following best practice recommendations [76]. A full list of safety measures can be viewed in Additional file 1: Item S1. Although involvement of the autistic community had not been possible beyond informal conversations between the primary researcher and a handful of autistic associates, this is a standard to which all research should aspire [63] and, given the opportunity to give feedback on the study, many important and helpful suggestions for future research were gleaned from participants.

### Analysis

The data for each variable and outcome measure were checked for outliers (Cook’s test), normality (skewness and kurtosis), autocorrelations (Durbin-Watson statistic), and visually checked for homoscedascity and normal distribution of residuals. Descriptive statistics were computed for suicide ideation in the previous year, lifetime suicide attempts, and recency of latest attempt. Subsequently, two lines of analysis were pursued.

#### ITS constructs as predictors of suicide ideation and attempts

While the ITS suggests main effects of thwarted belongingness, burdensomeness and acquired capability as predictors of suicide ideation and attempts, it also suggests additional variance is explained by the *interaction* of these states when they co-occur. To examine this, we performed a set of three regressions, correcting alphas level to *p* = 0.016 and setting confidence intervals at 95% in each case.

Two of these analyses were linear regressions, one for past-year suicide ideation (using our composite measure) and one for lifetime suicide attempts. In the regression for suicide ideation, the Enter method was used to model depression and anxiety in the first block of the model, to disentangle their effects as covariates. Given the hypothesised centrality of thwarted belongingness and burdensomeness for suicide ideation [14], and the proposed contributions of mental rehearsal to strengthening suicide ideation while building capability [36], these variables were entered in the second block of the model. To test the hypothesis that the interaction of thwarted belongingness and burdensomeness would explain greater variance than either variable alone [14], the interaction term of these variables was entered in the third block of the model, as per Pelton and colleagues [9]. This approach has been used widely [64, 65] to create a proxy for states where these ITS constructs co-occur at high levels. The second linear regression, for lifetime suicide attempts, modelled depression and anxiety in the first block; all factors of acquired capability, thwarted belongingness and burdensomeness (given their unexpected relevance to suicide attempts in Pelton et al.) in the second block; then, as before, the interaction term of thwarted belongingness, burdensomeness and acquired capability (total ACWRSS score) in the third block of the model.

The third regression was a confirmatory one, performed to address the problem that our index of lifetime suicide attempts gave no indication as to when suicide attempts had occurred and might, thus, be less relevant to ITS constructs measured in the present day. For a more time-sensitive index of past year suicidality, participants were categorised as having experienced suicide ideation, as having attempted suicide, or as having experienced neither suicide ideation or attempts. A multinomial regression was conducted, including thwarted belongingness, burdensomeness, each facet of acquired capability, and of course anxiety and depression as predictors. This confirmatory analysis is described in full in Additional file 1: Item S2.

In relation to all three regressions, multicollinearity checks indicated independence of ITS constructs from one another and from depression and anxiety (VIF ranged between 1.09 and 1.80, and tolerance between 0.56 and 0.94). Depression and anxiety were more closely related, but acceptably distinct (VIF 2.12, tolerance 0.47). With respect to the linear regressions, however, it is important to note that with the addition of the interaction terms, VIF and tolerance values indicated problematic multicollinearity. This is likely due to the way in which these interaction terms were computed, but is of note when interpreting the results.

#### ITS constructs as mediators of suicide risk associated with autism-relevant factors

Next, we examined the relevance of ITS constructs as mediators of suicide risk posed by two autism-relevant variables: specifically, of thwarted belongingness and burdensomeness as mediators of associations between suicidality and (a) relationship status, and (b) age at diagnosis. Prior to these analyses, point-biserial correlations were plotted between these two variables to confirm their independence from one another. We also examined their associations with age and sex, variables of relevance with respect to suicide. Greater age at diagnosis, in particular, was strongly associated with older age generally (*r* = 0.87, *p* < 0.001). As these variables could influence relationships between factors of interest, they were controlled for as covariates in each mediation analysis. These were performed using Model 4 from the PROCESS macro for SPSS [66] (version 3), a programme for mediation analysis which utilises ordinary least squares regression with bootstrapping (5000 samples). In each instance, confidence intervals were again set at 95%.

##### Relationship status

Relationship status was operationalised as a binary variable in which participants were coded as ‘single’ (0, including the 23 who were divorced, separated, or bereaved^1^) or in a relationship (1, including cohabiting, married, or dating/in a relationship); for this analyses, we removed the 10 participants who declined to answer this question or stated that they were asexual/aromantic. Two mediation analyses were performed with this predictor, with alpha levels corrected to *p* < 0.025 in each. The first examined direct and indirect effects of relationship status (the predictor, *X*) on the composite measure of suicide ideation in the last year (the outcome, *Y*) via the parallel mediators of thwarted belongingness, burdensomeness, depression and anxiety (which, closely related to suicide ideation, were included as mediators in their own right). Because relationship status is a binary variable which could have changed recently, it was deemed inappropriate to examine in relation to lifetime suicide attempts which could have occurred any time during a participant’s lifespan. Having previously modelled relationships between ITS constructs and past-year ideation, for completeness we examined whether relationship status was associated with increased likelihood of suicide attempts in the last year through its effects on suicide ideation as a mediator (Additional file 1: Item S3).

##### Age at diagnosis

Age at diagnosis, a continuous variable, was checked for normality and equality of variance at each level of the binary variables (e.g. sex, relationship status). The first of two mediation analyses with this variable modelled thwarted belongingness, burdensomeness, anxiety and depression as mediators, and suicide ideation as the outcome measure (*Y*). The second used lifetime suicide attempts as a continuous outcome measure (*Y*), and examined its relationship with age at diagnosis (*X*) through the mediators of thwarted belongingness, burdensomeness, depression and anxiety. As before, alpha levels were corrected to *p* = 0.025.

## Results

**Fig. 1:**
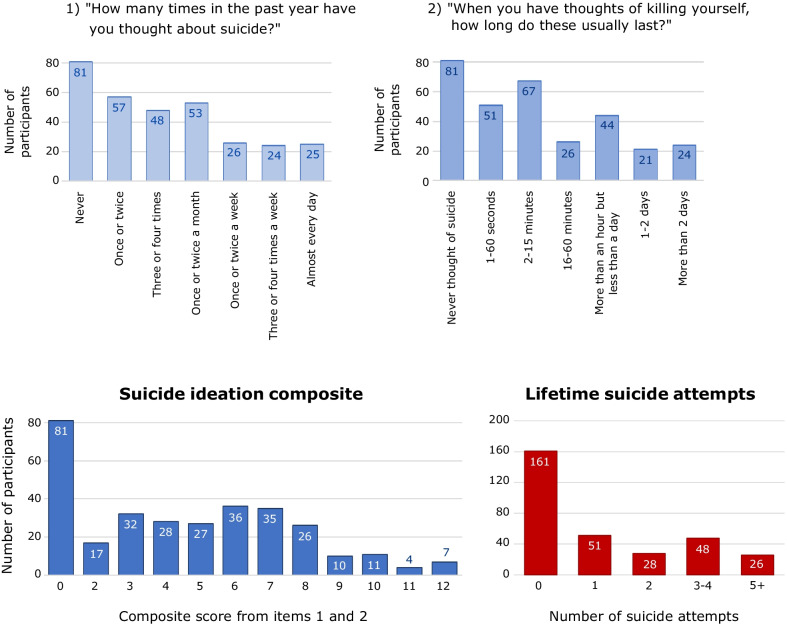
*Note*. Frequency of responses to the two items assessing past-year suicide ideation (top row). On the bottom row are displayed the frequency of scores in the suicide ideation composite and the number of lifetime suicide attempts endorsed by participants

The majority of participants (*n* = 233, 74.3%) reported some degree of suicide ideation in the past year. Within this group, a sizeable number thought of suicide on a weekly basis, and for sustained periods (see Fig. 1). The average score on our composite measure of past-year suicide ideation was 4.38 (SD 3.36).

Almost half the sample had a history of lifetime suicide attempts (*n* = 153, 48.7%). Twenty-eight (8.92%) had attempted suicide at least once in the last year; 67 (21.35%) reported that their last suicide attempt had been within the past 5 years; the average time since suicide attempts in this group was 10.59 years (SD 11.89), with the furthest back attempt 54 years ago.

### Relationships between suicide ideation, suicide attempts, and ITS constructs

The full model for past-year suicide ideation (including depression and anxiety [first block], thwarted belongingness, burdensomeness and mental rehearsal [second block], and the thwarted belongingness * burdensomeness interaction term [third block]) explained 53% of the variance in this outcome (*R*^2^ = 0.53, *F* [6, 313] = 57.10, *p* < 0.001). The addition of second-block variables significantly increased the explanatory power of the model, which had formerly explained only 36% of the variance in suicide ideation (*R*^2^ = 0.36, *F* [2, 313] = 88.94, *p* < 0.001; *R*^2^ change = 0.16, *F* change = 35., *p* < 0.001); addition of the interaction term, in the third step of the model, did not however explain a significantly greater amount of the variance than that associated with second-block variables (*R*^2^ = 0.53, *F* [5, 313]  = 68.43, *p* < 0.001; *R*^2^ change = 0.001, *F* change = 0.75, *p* = 0.387). While anxiety did not significantly contribute to predicting suicide ideation in any model, depression remained a distinct contributor to the variance in suicide ideation in the second (*B* = 0.15, *p* < 0.001; CI 0.09, 0.21; *r*^2^_part_ = 0.03) and full model (*B* = 0.14, *p* < 0.001; CI 0.08, 0.20; *r*^2^_part_ = 0.03). The second stage of the model also included significant contributions from burdensomeness (*B* = 0.10, *p* < 0.001; CI 0.07, 0.14; *r*^2^_part_ = 0.05) and mental rehearsal (*B* = 0.14, *p* < 0.001; CI 0.10, 0.18; *r*^2^_part_ = 0.08); the latter remained significant in the third stage of the model (*B* = 0.14, *p* < 0.001; CI 0.10, 0.18; *r*^2^_part_ = 0.08). Individual effect sizes (*f* ^2^) for each of these significant predictors, calculated on the basis of their squared semipartial (“part”) correlations, were all small (< 0.10).

For lifetime suicide attempts, the inclusion of ITS constructs (thwarted belongingness, burdensomeness and acquired capability facets) in the second step of the model explained 20% of the variance in number of lifetime suicide attempts (*R*^2^ = 0.20, *F* [7, 313] = 10.80, *p* < 0.001), a significantly greater proportion than that explained by depression and anxiety alone (*R*^2^ = 0.05, *F* [2, 313] = 8.30, *p* < 0.001; *R*^2^ change = 0.15, *F* change = 11.25, *p* < 0.001). The addition of the three-way interaction term (thwarted belongingness * burdensomeness * acquired capability total score) in the third block did not significantly contribute to the variance explained by the model (*R*^2^ change = 0.001, *F* change = 0.30, *p* = 0.588; *R*^2^ = 0.20, *F* [8, 313] = 9.46, *p* < 0.001). Although depression predicted suicide attempts in the first step of the model (*B* = 0.05, *p* = 0.005; CI 0.01, 0.08; *r*^2^_part_ = 0.02), it and anxiety were non-significant predictors after the addition of second-block variables. At this stage, predictors which significantly contributed to more numerous suicide attempts, albeit with small effect sizes (*f* ^2^ < 0.08), were burdensomeness (*B* = 0.03, *p* = 0.004; CI 0.01, 0.05; *r*^2^_part_ = 0.02), reduced fear of death (*B* = 0.05, *p* = 0.001; CI 0.02, 0.08; *r*^2^_part_ = 0.03) and mental rehearsal of suicide (just over the adjusted alpha level at *B* = 0.03, *p* = 0.019; CI 0.00, 0.05; *r*^2^_part_ = 0.01). The three-way interaction, in the third step, did not significantly predict lifetime suicide attempts, though associations with reduced fear of death (*B* = 0.05, *p* = 0.005, CI 0.02, 0.08; *r*^2^_part_ = 0.02) remained significant (a contribution of mental rehearsal remained above the adjusted alpha level at *p* = 0.035).

Our confirmatory multinomial regression on the basis of past-year suicidality (Additional file 1: Item S2) largely corroborated these results: participants who had attempted suicide were differentiated from non-suicidal participants by their higher scores in burdensomeness and their reduced fear of death (higher scores in mental rehearsal fell just above the corrected alpha levels). Participants who had attempted suicide differed from those who had experienced suicide ideation only on the basis of reduced fear of death (greater scores in burdensomeness and higher anxiety fell just above corrected alpha levels).

### Associations between autism-relevant factors and suicidality via ITS constructs

#### Relationship status

**Fig. 2:**
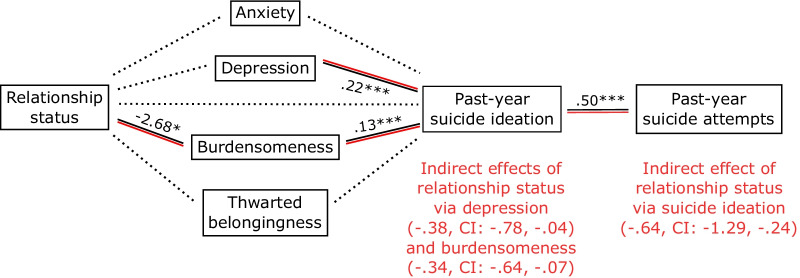
*Note*. Indirect effects of relationship status on past-year suicide ideation and attempts. The coefficients of associations significant below the corrected alpha level (.025) are marked by bold lines and a single asterisk (*); those significant at *p* < .001 are depicted by bold lines and three asterisks (***). Dotted lines reflect associations that were not significant

Relationship status was significantly associated with burdensomeness (*B* = − 2.68 (SE 1.09), *p* = 0.014, CI − 4.81, − 0.54), which was higher in those who were single; participants who were single also tended to be more depressed, though this did not remain significant after statistical correction (*B* = − 1.76 (SE 0.82), *p* = 0.0329, CI − 3.37, − 0.14). The model predicting suicide ideation (*R*^2^ = 0.49, F (7, 296) = 40.56, *p* < 0.001) was contributed to by depression (*B* = 0.22 (SE 0.03), *p* < 0.001, CI 0.15, 0.28), burdensomeness (*B* = 0.13 (SE 0.02), *p* < 0.001, CI 0.09, 0.16), and by the covariate of sex (where being male was associated with lower suicide ideation: *B* = − 0.86 (SE 0.33), *p* = 0.0085, CI − 1.51, − 0.22). The direct effect of relationship status on suicide ideation was non-significant (*B* = − 0.56 (SE 0.29), *p* = 0.0525, CI − 1.13, 0.01), but it was indirectly associated with suicide ideation via the mediators of depression (*B* = − 0.38 (bootSE 0.19), bootstrapped CI − 0.78, − 0.04) and burdensomeness (*B* = − 0.34 (bootSE 0.14), bootstrapped CI − 0.64, − 0.07). Relationship status was indirectly associated with past-year suicide attempts via its effect on suicide ideation (Fig. 2; see Additional file 1: Item S3 for statistical notations).

#### Age at diagnosis

Age at diagnosis was not significantly (*p* > 0.1) related to anxiety, depression, thwarted belongingness or burdensomeness, all of which were employed as mediators in models of past-year suicide ideation and lifetime suicide attempts. It was unrelated to both outcomes.

## Discussion

Following on from previous efforts [9, 33], the present study re-examined thwarted belongingness, perceived burdensomeness and acquired capability as *predictors* of suicide ideation and attempts in autistic adults, disentangling their main and interaction effects from those of depression and anxiety. As another means of exploring the relevance of these constructs, our second set of analyses explored their role as *mediators* of suicide risk. As being single and older age at diagnosis were previously associated with suicide deaths and attempts in autistic people [4], we examined whether these associations were, in fact, *explained* by higher levels of thwarted belongingness and perceived burdensomeness in these individuals. We will consider each set of analyses in turn, beginning with those which explored main effects and interactions derived from hypotheses of the ITS.

### The validity of ITS hypotheses in relation to autistic suicidality

The ITS conceptualises suicide ideation and suicide attempts as distinct phenomenon associated with specific proximal risk factors [12, 14]. For suicide ideation, it asserts the relevance of thwarted belongingness and burdensomeness, with recent suggestions that mental rehearsal, a facet of acquired capability, may also contribute unique variance [36]; for suicide attempts, it purports the especial relevance of acquired capability for suicide. Statistically, the *relevance* of these factors should be reflected in main effects of each predictor on these outcome variables, main effects which should, importantly, be independent of any contributions of depression and anxiety [35]. An additional, important tenet of the ITS posits that the *interaction* (combination) of thwarted belongingness and burdensomeness is necessary to transform ‘passive’ to ‘active’ suicide ideation, and that likewise, that suicide attempts will only occur in the presence of active suicide ideation (thwarted belongingness + burdensomeness) *and* capability for suicide. Statistically, this suggests that interaction terms reflecting co-occurring levels of each state should explain unique variance in models of suicide ideation and suicide attempts. With these theory-derived predictions in mind, we begin by interpreting our data around suicide ideation.

With regards to the *relevance* of ITS constructs for suicide ideation, we observed main effects of burdensomeness and the mental rehearsal aspect of acquired capability, over and above effects of depression (anxiety did not significantly contribute to the model). Altogether, variables included in the model explained substantial variance in suicide ideation (53%, as compared to 10% in Pelton et al.); the difference may reflect our inclusion of depression and mental rehearsal and our operationalisation of suicide ideation, which was slightly more time-sensitive (focusing only on the past year) and, based on the composition of two items, more indicative of severity/frequency.

While the lack of association with thwarted belongingness was contrary to the assertions of the ITS [14], it is in fact consistent with a number of studies purporting greater relevance of burdensomeness over thwarted belongingness in the general population [67–70]. The relevance of burdensomeness for suicide ideation and furthermore *attempts* in our sample, even while their average score (17.1) fell below the cut-off [22] proposed for optimal sensitivity and specificity in the general population [71], suggests it may be a potent contributor to suicidality. At the same time, given that our participants scored well above the suggested clinical cut-off [31] for thwarted belongingness, our findings cast doubt on the relevance of this variable, which not only failed to predict suicide ideation (or attempts) but failed to differentiate non-suicidal participants from those who had experienced suicide ideation and attempts. Though they *did* observe relationships between thwarted belongingness, suicide ideation and attempts [9], Pelton and colleagues suggest that feelings of thwarted belongingness may be far more “typical” of social experiences in autistic people and more reflective of pathological social disconnection in non-autistic people [72]. It is possible that in our study, items of the INQ-15 failed to capture the more noxious forms of these experiences and feelings in autistic people; previous research does suggest that autistic and non-autistic respondents interpret these items differently [72]. Accordingly, abandonment of this construct as a suicidogenic factor would be premature, especially given the recognised contributions of loneliness to mental illness, self-injury and suicidality in autistic people [6, 73].

In addition to examining the individual relevance of thwarted belongingness and burdensomeness to suicide ideation, our study aimed to examine additional risk conferred by the two-way *interaction* or combination of these two states, an important element of the ITS. In this aspect, our findings corroborated those of Pelton and colleagues [9] in opposing this particular hypothesis as it pertains to autistic adults. Both studies computed interaction terms which were assumed to mark out participants who were experiencing strong feelings of both thwarted belongingness *and* burdensomeness. The multicollinearity induced by addition of the interaction term obscures any estimation of the unique variance apportioned to it, but the fact that there was no significant change in the variance explained by the model suggests that the combination of these two factors was no more potent than that of either construct alone. This is in fact not inconsistent with extant literature from the general population, as most studies fail to support the interaction hypotheses of the ITS [64, 65]. The emergence of relationships between ITS constructs and indices of suicidality is notoriously sensitive to the operationalisation of the variables in question [65], and the interaction hypotheses are particularly difficult to test in that they concern the alignment of dynamic, fluctuating states. We suspect that rather than constituting valid evidence against this particular ITS hypothesis, the statistical interactions computed in the present study and by Pelton et al. were simply unrepresentative of real, dynamic “interactions”, in the non-statistical sense of the word, between co-occurring thwarted belongingness and perceived burdensomeness. With both cross-sectional in design, it is likely that neither was temporally precise enough to capture the theorised contingency between these fluctuating states and suicidal outcomes. In addition, neither were we able to make the fine-grained distinction asserted by the ITS wherein the combination of burdensomeness and thwarted belongingness transforms passive wish for death (the result of either of these variables alone) into active suicide ideation.

As such, given this potential experimental failure to operationalise the co-occurrence of these states, we must refrain from strong conclusions as to the validity of this ITS interaction hypotheses in autistic samples; in relation to suicide attempts, it is likely that the same explanation accounts for why neither our study, nor that of Pelton et al., observed unique variance explained by the three-way interaction between thwarted belongingness, burdensomeness and acquired capability. Where both studies agree, however, is that the trajectory of suicide ideation to attempts, in autistic people, might differ to that described by the ITS. Given that perceived burdensomeness contributed to both suicide ideation *and* suicide attempts in both studies, this casts doubt on the separability of factors involved in the two phenomena and suggests that constructs canonically understood to predict suicide ideation might, in fact, be sufficient to motivate the transition to suicide attempts in autistic people. In relation to suicide attempts, we turn now to the least researched and most debated construct of the ITS [29, 64, 74, 75]: acquired capability for suicide.

Modelled alongside thwarted belongingness, burdensomeness, anxiety and depression, the model including this broader conceptualisation of acquired capability explained slightly more of the variance in lifetime suicide attempts than that seen by Pelton and colleagues (20%, as compared with 15%); in our confirmatory multinomial regression, these factors together explained 55% of the variance in past-year suicide attempts. Our scale, the ACWRSS, is based on a three-factor model comprising heightened pain tolerance, reduced fear of death, and mental rehearsal of suicide plans [36]. While acquired capability is generally associated with suicide attempts over ideation, this approach suggests that mental rehearsal of suicide plans accrues capability for suicide while also strengthening the desire for death [36]—consistent with its main effects on both suicide ideation and lifetime suicide attempts in our sample. Mental rehearsal may be partially synonymous with “suicide-specific rumination” [76, 77], which in recent publications appears similar in respect to contributing to both ideation and action. In our multinomial regression, scores in mental rehearsal could differentiate non-suicidal participants from those who had attempted suicide in the past year, but could not differentiate participants with past-year experience of suicide ideation from those with experience of suicide attempts.

The other two facets of acquired capability, reduced fear of death by suicide and heightened pain tolerance, operated only partially as hypothesised by the ITS. Reduced fear was associated with more numerous lifetime suicide attempts, and differentiated those who had attempted suicide in the past year from those who were non-suicidal and those who had experienced past-year suicide ideation. This is somewhat comparable to Pelton et al. [9], who, using the ACSS-FAD, observed reduced fear in autistic and non-autistic individuals with at least one historical suicide attempt. These authors also observed an impact of lifetime trauma on suicidality via reduced fear of death (though this relationship was attenuated in autistic individuals). While this is in apparent support for the ITS, we cannot establish causal primacy of reduced fear of death over suicide attempts. This serves as a poignant reminder of an inherent experimental limitation of conducting research in this sphere: that we can only include people who have survived suicide attempts in research, who might subsequently, or consequently, have reduced fear of dying. Prospective longitudinal studies with large samples (given that suicide occurrences are, thankfully, still comparatively rare) are required to explore whether reduced fear of death indeed creates capability for suicide attempts in the manner suggested by the ITS.

With regards to the other facet of acquired capability, higher pain tolerance was not associated with lifetime or past-year suicide attempts in our sample. This may be due to limitations of the ACWRSS, which includes only 2 items for pain tolerance. To date, there is no clear consensus as regards the assessment of acquired capability and, relatedly, its factor structure [75]; the ACWRSS was flagged as an alternative to earlier scales with weaker psychometric properties [74], but has not received vigorous psychometric scrutiny beyond the claims of the original authors, who asserted its “excellent fit” in two independent non-autistic samples [36]. Although the scale itself might be at fault, an alternative interpretation is that the scale, and/or this facet of acquired capability, simply functions differently in autistic people. Pain tolerance may be less relevant for suicidality, as might be expected if pain perception is atypical [78]—indeed, even in the general population the importance of this facet has been queried [75]. A broader view of “suicide capability”, one that might encapsulate additional factors, such as dispositional features, practical knowledge and means [75], may be valuable in autism. While the static snapshots provided by these cross-sectional studies suggest the significance of mental rehearsal of suicide plans and reduced fear of death, future longitudinal investigation should also consider the likely disparities in temporal stability within the facets comprising suicide capability [79, 80], along with the proximal factors which evoke suicidal desire and intent in individuals with the means to enact them.

### Risk factors within the autistic community

To maximise the explanatory power and applicability of theories developed in the general population, there is a need to consider how these hypotheses might relate to characteristics or factors relevant to an autistic population [81]. As such, to further explore the relevance of ITS constructs in autistic people, we examined the role of thwarted belongingness and perceived burdensomeness as *mediators* of suicide risk associated with certain factors. (Acquired capability did not appear theoretically relevant to the factors in question, hence its exclusion from this set of analyses). Since the literature in autism has highlighted relationships between suicidality, loneliness and lack of social and practical support [6, 7], we first focused on (romantic) relationship status. Given that intimate relationships are often a key source of emotional, social and practical support [37], the attenuation of suicide risk in autistic individuals who were married or cohabiting [4] appears intuitively logical—and, we hypothesised, might be underpinned by reduced feelings of thwarted belongingness and burdensomeness. Relationship status (being in a relationship) was indeed associated with lower likelihood of past-year suicide ideation indirectly via reduced feelings of burdensomeness and depression, and was associated with lower likelihood of past-year suicide attempts via this reduced likelihood of suicide ideation. As burdensomeness (as conceptualised by the ITS) incorporates both “affectively-laden cognitions” of self-hate, and feelings of being a burden or liability to others, it is conceivable that relationship status provides affirmations of worth which (to some degree) ameliorate self-hate.

It is likely, however, that factors uncontrolled for in this analysis, such as employment status, health status and living situation, are important determinants of burdensomeness, and may have also differentiated participants in relationships from those who were single. Surprisingly, our results indicated that individuals in romantic relationships were no less likely to experience feelings of thwarted belongingness; it is, however, important to note that while some INQ-15 items reflect daily interactions, others reflect broader feelings of social connectedness or lack thereof (“These days, I feel like I belong”, “… I often feel like an outsider”). As a construct, thwarted belongingness encapsulates loneliness and social isolation but extends in a wider sense to “the need to belong”, to care for others and feel cared about [14]. Unconditional regard or acceptance of an individual’s authentic self would seem an inherent element of such care, which may explain why some studies report associations between thwarted belongingness and minority status [82, 83], and why camouflaging autistic traits is associated with suicide ideation via thwarted belongingness [84].

Our binary coding for relationship status lacked nuance around relationship satisfaction, and as such, we cannot infer whether participants received this kind of acceptance within their close relationships, or if any such acceptance from close others was sufficient to ameliorate feelings of wider social disconnection and marginalization [22, 85]. This analysis corroborated the aforementioned importance of burdensomeness in suicide ideation, but future research may benefit from deconstructing both thwarted belongingness and burdensomeness as constructs, distilling both to understand relationships between these more fine-grained factors (e.g., self-hate) and suicide risk, and the relative importance of how autistic people perceive their close and wider relationships with the community around them.

The second autism-relevant factor of interest in this study was age at diagnosis. Given the association between later age at diagnosis and depression, self-injury and camouflage [40, 41, 43, 48], and the recently observed elevation of suicide attempts and deaths as age at diagnosis increased [4], we hypothesized that later age of diagnosis might be associated with heightened feelings of burdensomeness, thwarted belongingness, depression and anxiety, and through these, increased likelihood of suicidality. In fact, it was not significantly associated with any one of these mediators, or with suicide ideation or attempts. This is consistent with one previous study which likewise observed no relationship between increased age at diagnosis and suicidality [6]. While this might be perceived as reassuring, we suggest this null finding may be more likely due to our operationalization of and lack of variance in this variable, since most of our participants were diagnosed in adulthood.

While we treated age at diagnosis as a continuous variable, previous studies performed group comparisons of younger and later-diagnosed autistic youth [40, 41]; it is possible that effects are more apparent when examined categorically, and that once an individual passes a certain age, differences between diagnosis at age 20 vs. 30, for instance, are minor in comparison to differences between diagnosis at age 5 vs. 15. Given the discord between the present findings and those seen in teenagers and children [40, 41], it is possible that effects of late (or missed) diagnosis are more influential at some parts of the lifespan than others, and at this point, dimensional effects of late diagnosis are not prominent given how common mental health problems are in the adult autistic community. These are important questions for future research.

## Limitations and future directions

There are a number of important limitations of this study, foremost the cross-sectional nature of our design. Thwarted belongingness, burdensomeness and acquired capability are conceptualised as dynamic, fluctuating states, and suicidal behaviour, according to the ITS, is contingent on the alignment of proximal factors (thwarted belongingness and burdensomeness) with presently high capability: the suicide wish *and* the ability to act on it [14, 64]. Though we purported to examine the interaction hypotheses of the ITS, we suggest it highly probable that the present study failed to adequately operationalise this aspect of our investigation. The practical and ethical challenges of experimental research in suicidality are substantial [86], but it is important that research on suicide in autism recognise limits to the implications which can be drawn from our work, particularly when drawing associations between measurements of present-day states and indices of lifetime suicidality. The present study operationalised an index of past-year ideation and past-year attempts in order to, as closely as possible, match present mental states with recent suicidal behaviour. We are able to report statistical associations suggesting that burdensomeness, mental rehearsal of suicide plans and reduced fear of death may be especially relevant to past-year suicide ideation and attempts, but further than this, cross-sectional designs are inadequate for testing dynamic interactions between suicidogenic risk factors as per the ITS.

This is additionally true because we cannot establish causality or directionality in any of the associations observed. For instance, given that depression generates interpersonal problems [87] and thwarted belongingness and burdensomeness mediate this relationship [88], it is possible that by extension, suicide ideation and attempts were deleterious to the close relationships of our participants, preceding or at least strengthening the development of thwarted belongingness and burdensomeness. Depression and anxiety were treated as covariates in our primary analysis due to their purported subordinance to thwarted belongingness and burdensomeness [14]; this statistical approach is common in ITS studies [35] but not beyond scepticism, given its assumption that thwarted belongingness and burdensomeness are the mediators between depression and suicide ideation as opposed to the other way around [89].

Furthermore, since non-fatal suicide attempts are understood to increase capability for further attempts [90], we cannot ascertain whether past-year suicide attempts preceded participants’ self-reported fearlessness about death, mental rehearsal, or pain tolerance after the fact. Ideally, investigation of ITS hypotheses should be embedded in longitudinal designs, as have been imaginatively enacted in suicide research [68–70, 80]. Partnership with the autistic community will be vital in balancing risk and scientific rigor in this challenging field [91].

Although our primary aim was to examine whether ITS constructs bore relevance for autistic participants, an age- and IQ-matched comparison group within this study would have shown, more clearly, where differences in the relevance and strength of relationships might occur. As it pertains to the relevance of ITS constructs in autism, a related issue lies in the assumption that normative assessment tools for ITS constructs (and indeed, anxiety) operate similarly in autistic people. The internal consistency of our scales was good, but concerns have been raised that people high in autistic traits might under-report thwarted belongingness [84], and autistic people have been seen to respond differently to non-autistic people on some items of the INQ-10 [72]. We cannot presently ascertain with certainty whether ITS constructs are more or less relevant in autism or simply insufficiently operationalised, and as such support the assertions of Pelton and colleagues [72] around the need for in-depth and mixed methodological scrutiny of these constructs, their meaning, interpretation and relevance, in autism.

There are a number of limitations related to our sample which bear relevance for future research efforts. Firstly, while all participants reported the place and date of their diagnosis, we did not independently verify these. While the majority of participants were British, we cannot testify to the standardisation with which these diagnoses were made across the UK, or indeed across the countries that participants hailed from. Given that 95% of the sample were British, and that nearly 80% described their ethnicity as Caucasian/White, our findings must be recognised as culturally specific and not necessarily representative of autistic people from other countries and with other ethnic backgrounds.

The generalisability of our sample to the wider autistic population is limited in other ways. Firstly, our methods can be assumed to have excluded participants with severe communication difficulties or intellectual disability, being accessible only to those who were able to see and respond to an online advertisement. While we may assume that none of our participants had the kind of profound cognitive impairment that would preclude their participation, we did not assess cognitive or intellectual abilities, which can be reasonably assumed to modify suicidal thoughts and behaviour [92, 93] and which might, indeed, have contributed to variation in our sample. Cognitive abilities intersect with language abilities, but given that there are many other reasons why some autistic people are non-speaking or use minimal spoken language [94], the present study failed to ascertain whether all participants used verbal communication. This, too, may have been an important moderator of an individual’s social world, and particularly relevant to the ITS. Both autistic adults with intellectual disability *and* those who are non-speaking are underrepresented in scientific literature, necessitating attention to these underserved groups.

Our advertisement emphasised that all volunteers were welcome, including those who had never been suicidal or self-injured, but it is possible that participants with more vested interests in these areas (i.e. poorer mental health) may have self-selected, although we did not highlight which risk factors (predictors) were our focus. As in Pelton et al. [9], our sample was heavily skewed towards autistic women, who we have previously speculated [8] may be more willing than autistic men to take part in studies about mental health, just like their neurotypical counterparts. We chose to control for effects of sex and age, but it is entirely possible, as in normative populations [65, 95], that these features moderate the strength and significance of relationships between ITS constructs and suicidality. Other variables of potential relevance include broader psychopathological symptoms (e.g. of PTSD, OCD and alcohol abuse disorder), which may contribute additional explanatory power when modelled alongside ITS constructs [96]. The relationship between marginalised gender identities and mental health is a community priority [91] and the intersectionality of gender non-conformity and autism would also be expected to bear relevance to ITS constructs [82, 83].

While the present study contributes to a body of literature highlighting variables of putative relevance for suicide risk, it is important to consider other autism-related cognitive factors, including black-and-white binary thinking (e.g., “life is/is not worth living”), difficulties imagining the perspectives of others (e.g., “my family will be happier without me”), intense focus and fine-grained attention to detail (e.g., excessive researching methods of suicide), as well as social and societal factors such as vulnerability to victimisation [17] and social isolation. Future studies should also consider factors which, if nurtured, might ameliorate suicide risk, such as belief in coping ability [97] or connectedness with family and friends [98]. Individual-level interventions must, however, be auxiliary to societal efforts to reduce prejudice and discrimination suffered by autistic people, given the role of autism-related stigma in camouflage [99] and poor mental health [22], and the association between marginalisation and suicide seen in other groups [82, 100].

Finally, from a theoretical standpoint, it is vital that future research remains open to other major perspectives in suicidality [29], and considers these with respect to their temporal dynamics and situation within suicide trajectories. Fluid vulnerability theory [101] suggests that suicide risk is non-linear, with individuals liable to influences from static, historic or largely stable risk factors (e.g. race, genetics, gender, disposition) and state-based, acute ones influenced by the present environment (e.g. mood, life stress, hopelessness). The theory suggests that the presence of numerous stable factors creates a higher baseline risk level from which individuals can move rapidly into suicide crises, more likely to be triggered by a wider number of stressors in the environment. Although uninvestigated in autism , a foundational assumption that autistic individuals may be at higher baseline risk appears consistent with higher rates of suicidality and may be a useful base point for future studies.

## Conclusions

Our data suggests that feelings of burdensomeness may be a potent factor in suicide ideation *and* attempts, and that facets of acquired capability, notably reduced fear of death and mental rehearsal of suicide plans, may likewise contribute to both ideation and attempts. Our analyses indicate that these associations may underpin some of the increased risk associated with variables like relationship status, which justifies further investigation of how ITS constructs are conceptualised and how they should be measured in autism. In collaboration with the autistic community, further research must devise safe and scientifically rigorous methods to examine these associations in longitudinal, time-sensitive designs.

## Supplementary Information

**Additional file 1.** Additional details of methods and analyses.

## Data Availability

The datasets used and/or analysed during the current study are available from the corresponding author on reasonable request.

## Abbreviations

ACWRSS: Acquired Capability with Rehearsal for Suicide Scale
GAD-7: Generalised Anxiety Disorder-7, a measure of generalised anxiety
INQ-15: Interpersonal Needs Questionnaire, a measure of perceived burdensomeness and thwarted belongingness
ITS: Interpersonal Theory of Suicide
PHQ-9: Patient Health Questionnaire-9, a measure of depression
SITBI: Self-Injurious Thoughts and Behaviours Interview

## Footnotes

1 Bereaved individuals (n = 4) might be considered to constitute quite a different psychological profile than those who were divorced or separated, but were included in the ‘single’ category for simplicity. Analyses were checked without these individuals: the results did not change significantly.
