# Correction to: The relevance of the interpersonal theory of suicide for predicting past-year and lifetime suicidality in autistic adults

**DOI:** 10.1186/s13229-022-00496-4

**Published:** 2022-03-28

**Authors:** R. L. Moseley, N. J. Gregory, P. Smith, C. Allison, S. Cassidy, S. Baron-Cohen

**Affiliations:** 1 Department of Psychology, Bournemouth University, Talbot Campus, Fern Barrow, Poole, BH12 5BB Dorset UK; 2 Autism Research Centre, Department of Psychiatry, University of Cambridge, Cambridge, UK; 3 School of Psychology, University of Nottingham, Nottingham, UK

## Correction to: Molecular Autism (2022) 13:14 https://doi.org/10.1186/s13229-022-00495-5

Following publication of the original article [1], the authors reported missing information in the ‘Acknowledgements’ section. The corrected ‘Acknowledgements’ section reads:

We thank our participants for their generosity in taking part in the present research and for their helpful feedback towards this and future research. We would like to thank the ACORN panel at Bournemouth University for the funding this research, and our supportive colleagues. Special thanks goes to Rebecca Ellis for her patience in assisting with participant payments. We thank our colleagues at the Autism Research Centre who helped us recruit through their participant panel; the staff at Autistica who allowed us to advertise to their research network; moderators and owners of Facebook groups who were willing to let us advertise our study. Dr. Moseley thanks Dr. Sarah George, Dr. Helen Bolderston, and Dr. Cécile Bardon for their advice around ensuring the safety of participants, with especial thanks to Dr. Bardon for her warm encouragement. Finally, the authors remember all autistic people whose lives were lost to suicide, both within our participant cohort and the broader autistic community.

The original article [1] has been updated.
