# Supplementary material for: The relevance of the interpersonal theory of suicide for predicting past-year and lifetime suicidality in autistic adults
Source: Mol Autism. 2022 Mar 21;13:14. doi: 10.1186/s13229-022-00495-5 (PMC8935684; doi:10.1186/s13229-022-00495-5)
Supplement: Supplementary file 1 — Additional file 1. Additional details of methods and analyses. [file 13229_2022_495_MOESM1_ESM.docx]

**Additional file**

Item S1: Safeguarding measures

Item S2: Multinomial regression of past-year suicidality

Item S3: Indirect effect of relationship status on past-year suicide attempts via suicide ideation

Supplementary item 1: Safeguarding measures

Having sought guidance from a Clinical Psychologist and two researchers in autistic and in general-population suicidality, the following steps were taken to ensure participant safety and comfort while taking part in this high-risk study.

*Pre-emptive measures:*

1. All study documentation was visually augmented with simple icons for accessibility and clarity, and a picture of the primary researcher and her dog was provided to give a human and approachable face to the research team. An example as follows:


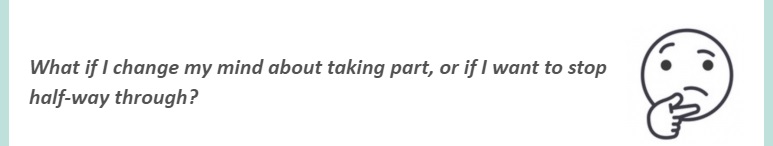


1. The Information Sheet, alongside informing participants of the nature of study, gave examples of the most provocative items in the survey.
2. Participants were asked to take time to consider carefully whether they wished to take part in the study and talk about this with their friends/family if so desired. They were encouraged not to take part if they thought that this topic might be upsetting for them at present, though assured of their ability to stop at any point once the study commenced.
3. Participants were assured that if they elected to take part, they were free to complete the questionnaires at their own speed, to take breaks, and to withdraw from the study at any point without penalty or needing to explain why. The survey was configured such that participants could complete the study over up to 14 days, and could log in and out of the study with the online server saving their progress as they went alone.
4. A coloured box on the information sheet provided participants with 7 ways to look after their mental health during and after the study, e.g.:


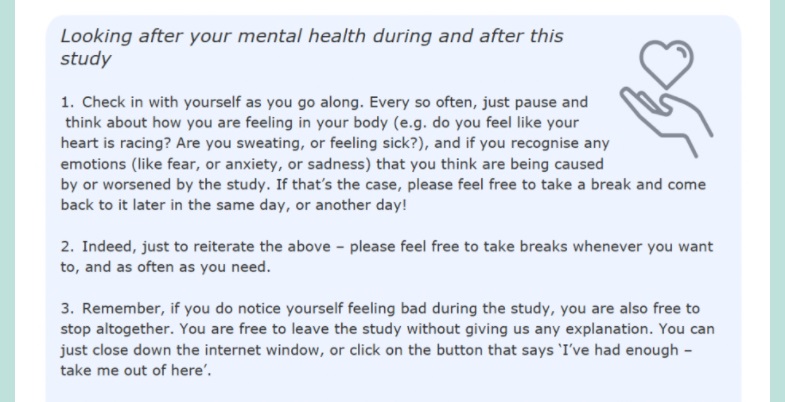


1. Signposting on the information sheet included one 24/7 phoneline (Samaritans) and four other phonelines (Papyrus, Sane, Supportline, CALM); three text services (Papyrus, SHOUT, and Crisis Textline); two webchats (CALM, Suicide Stop); a list of online resources including webtools and apps designed for crisis management from Rethink Mental Illness and Mind; a list of international services (international suicide hotlines, Befrienders Online); autism-specific resources (Autism Mental Health Awareness; The Thinking Person’s Guide to Autism, the National Autistic Society, Autistica); and a number of books on mental health written by and for autistic people.
2. Participants were also encouraged, on the information sheet, that if they were upset at any point during the study, they should speak to someone close to them, who would want them to call at any time; to talk to a medical professional or their GP; and, if they felt in immediate danger of hurting themselves, to call the emergency services.
3. Participants were provided with the link to a crisis plan worksheet (Mental Health Autism, Coventry University) which they might benefit from exploring, with their loved ones, at a later time.
4. Participants were also given a dedicated email address that they could use if upset by the topics of the study. It was emphasised that this was not an emergency contact and that the researchers were not clinically trained to support them through their feelings, but the researchers would respond as quickly as possible and would be happy to signpost participants to relevant services.
5. Participants could not proceed to the study unless they explicitly indicated their understanding of the nature of the study.

*During the study:*

1. Every page with a distressing questionnaire had a visual icon that participants could click to escape the survey. Doing so would take them to a page with all the support resources listed above, including the crisis plan by Mental Health Autism, which would remind them to reach out to those around them and potentially medical/emergency services if they needed to. All of this information was downloadable.
2. Accessible page-design was employed as per academic recommendations (Yu et al, 2018, *SMU Data Science Reviews*), and per the recommendations of the National Autistic Society: a mild-coloured background was used around a white box containing the text of the study; icons were used sparingly and simplistic in design; content was clearly separated by white space, spread out in an extended manner; text was likewise presented with extended spacing between characters, words, and sentences; the design remained consistent on each page; clearly contrasted coloured font, designed not to clash with the background colour, was used to highlight key terms.
3. The study was split into three parts, with corresponding mood-mitigation at two break-points and on the last, debriefing page. Cute and funny animal pictures displayed on these pages asked the participants to check in with how they were feeling, reminded them that it’s fine to take a break and come back to the study later, OR to stop for good if they’ve had enough. These pages also offered a simple mindfulness exercise (Five senses) that participants could undertake if they felt overwhelmed.
4. In addition to the two break-point pages, pages were inserted before the questionnaires on painful and provocative experiences, self-injury and suicidality. These pages, which included a mood-mitigating picture that was sweet and peaceful (duck and ducklings), explained humanely what questions were coming up, and explained why we needed to ask these questions, even though they might be upsetting to think about. Participants were reminded that they could leave the study at any time, including now if they felt they’d rather not go ahead with those questions, and were encouraged: “Please stay safe and put yourself first.”
5. Every page of the survey was presented in a warm, accessible style, with explanation of what each questionnaire measured and simple icons to aid comprehension.

*After the study:*

1. The debrief page of the study, which could be downloaded, included a couple of mood-mitigating items (a funny picture and video). The debrief sheet included all of the organisations listed above, and the apps, and reminder about emergency services. The contact details of the primary researcher were given again, along with the reminder to talk to loved ones, supporters or health workers if the person was struggling.
2. Participants were asked to enter their email address for the purpose of payment and so that, as the Information Sheet explained, we could contact them if we had concerns about their safety. Because participants took part anonymously, we explained that the system was configured to contact them automatically after they had completed the study. This occurred if responses to the PHQ-9 and suicidality items suggested that the participant was at high risk of suicidality. This triggered an email to be sent: this was a humane message from the primary researcher, explaining that their answers had given us concern and that we were genuinely sorry for their distress. Participants were reminded to call the emergency services if they felt in immediate danger, and that they should talk to their GP or health workers, and any friends and family who could help. They were encouraged to access the support resources previously described, and to look at the crisis plan from Mental Health Autism, which could be used as the basis for discussion with their loved ones.
3. The last page of the study also invited participants to ‘Have your say!’ – to tell us anything they liked or did not like about the study, and anything they felt we’d missed, if they so desired.

Although we cannot statistically or longitudinally testify to the efficacy of these steps, Autistica received positive feedback about the study from the members of their Research Network and many participants left appreciative feedback. A sample of this is as follows:

- “Thank you for looking into this issue. Best questionnaire I have ever completed – excellent questionnaire format. Excellent use of images. Clear layout. Love the photographs. Explanatory, friendly, ‘approachable’ text. Loved the intro and the picture of the researcher! Stress was massively reduced.”
- “The study is written with great sensitivity and obvious care for its participants, thank you”
- “A beautifully put together survey. Questions were easy to answer and I like that they are done in smaller sections, which means not having to keep scrolling back and forth to answer them. I like that you included cute pictures to help calm down after answering difficult questions. I felt like the author completely understood and showed high levels of empathy in the way the questions were asked and everything was written.”
- “I liked the layout of the study, easier to digest, follow and absorb the information required. A lot of thought has gone into it, just wanted to say thanks”
- “I liked the spaces between the questions. I liked being able to see the descriptive responses above and below the questions as I scrolled through. I liked the black and white pictures that went along with the questions. I liked seeing the animal photos as well.”

Supplementary item 2: Multinomial regression of past-year suicidality

**Rationale:** This confirmatory analysis followed on from the linear regression of lifetime suicide attempts, in which depression and anxiety were entered as a first step, and the facets of acquired capability, thwarted belongingness and perceived burdensomeness as a second step. The rationale for this multinomial regression was that lifetime suicide attempts as an index gave no indication of the recency of suicide attempts, which might thus bear limited relevance to ITS constructs measured in the present day.

**Method:** For the multinomial regression, participants were classified as non-suicidal if they had neither experienced suicide ideation nor attempted suicide in the past year, as having experienced suicide ideation in the past year, or as having made one or more suicide attempts in the past year. Participants categorised as having experienced suicide ideation were those who scored 4 or higher in the suicide ideation composite; those who scored 2 in the composite measure of suicide ideation (suicide ideation which had occurred once or twice in the past year and for 1-60 seconds) were categorised as non-suicidal. For a conservative approach, individuals who scored 3 were included in the suicide ideation category only if they had scored 2 on the duration question (suicide ideation which had occurred once or twice in the past year but for 2-15 minutes), but not if their suicide ideation had occurred 3-4 times in the past year but for less than 60 seconds each time. The presence of one or more suicide attempts in the last year classified participants in the suicide attempting group (the reference category), regardless of their scores in suicide ideation. Predictors in this model included depression, anxiety, thwarted belongingness, burdensomeness, and all facets of acquired capability. In addition to the previous checks, the data were examined for linearity between continuous predictors and the logit transform of our categorisation variable.

**Results:** Almost 77% of participants (76.4%) were correctly categorised by the model as non-suicidal (n = 111), as having experienced past-year suicide ideation (n = 175) or having made one or more suicide attempts in the past year (n = 28) - a significantly greater proportion than the intercept model alone (χ^2^ = 192.69, p < .001, Nagelkerke R^2^ = .55). Variables which significantly contributed to the model were depression (χ^2^ = 13.08, p = .001), burdensomeness (χ^2^ = 32.54, p < .001), reduced fear of death (χ^2^ = 10.22, p = .006), and mental rehearsal (χ^2^ = 24.95, p < .001). With the suicide attempting group as the reference, more specific differentiation between this group and non-suicidal participants showed that the latter tended to have lower scores in burdensomeness (B = -.19, Wald χ^2^ = 23.97, p < .001, OR = .83, CI: .77, .89), lower scores indicating that they were less likely to have lost their fear of death (B = -.18, Wald χ^2^ = 9.50, p = .002, OR = .84, CI: .75, .94), and lower scores in mental rehearsal, though these did not remain significant after correction (B = -.12, Wald χ^2^ = 5.40, p = .020, OR = .89, CI: .81, .98). The significant differences between participants who had experienced suicide ideation in the past year and those who had made suicide attempts were reduced fear of death in participants who attempted suicide (B = -.11, p = .017, OR = .89, CI: .81, .98), and just above the adjusted alpha levels, greater burdensomeness (B = -.07, p = .024, OR = .94, CI: .89, .99) and greater anxiety (B = -.12, p = .028, OR = .89, CI: .80, .99). Though significantly contributing to the overall model, depression scores did not significantly differentiate non-suicidal participants from those who had made suicide attempts, or differentiate those who had experienced suicide ideation from those who had made attempts.

Supplementary item 3: Indirect effect of relationship status on past-year suicide attempts via suicide ideation

For completeness, having modelled relationships between relationship status, ITS constructs and past-year suicide ideation, we examined indirect effects of relationship status on past-year suicide attempts via suicide ideation (as a mediator). Alpha levels were corrected to p = .025.

In this model of past-year suicide attempts (χ^2^ = 48.52, p < .001, Nagelkerke R^2^ = .33), relationship status exerted an indirect effect via suicide ideation (B = -.64 (bootSE: .27), BootCI: -1.29, -.24), which itself directly predicted attempts (B = .50 (SE: .09, p < .001, CI: .32, .68) (see Figure 2).
